# The Chinese translation and cross-cultural adaptation of PRISMA-7 questionnaire: an observational study to establish the accuracy, reliability and validity

**DOI:** 10.1186/s12877-024-04825-5

**Published:** 2024-02-28

**Authors:** Meredith T. Yeung, Yen Gan, Shu Qi Teo, Kai Quan Lim, Hui Xuan Leow, Myriam Jbabdi, Michel Raiche, Mingxing Yang

**Affiliations:** 1 Health and Social Sciences Cluster, Singapore Institute of Technology, 10 Dover Drive, Singapore, 169683 Singapore; 2 Centre d’expertise en santé de Sherbrooke / Sherbrooke Health Expertise Centre, 500, rue Murray, Sherbrooke, Québec, J1G 2K6 Canada; 3 Faculté des sciences de l’activité physique, Université de Sherbrooke, 2500 Boul. Université, Sherbrooke, Québec, J1K 2R1 Canada; 4 Singhealth Polyclinic, SHP-Head Office, 167 Jalan Bukit Merah Connection One (Tower 5), #15-10, Singapore, 150167 Singapore

**Keywords:** Aged, Frailty, Validity of case-finding tools, Geriatric assessment, Cross-cultural adaptation, Chinese

## Abstract

**Background:**

Frailty is a health condition linked to adverse health outcomes and lower life quality. The PRISMA-7, a 7-item questionnaire from the Program on Research for Integrating Services for the Maintenance of Autonomy (PRISMA), is a validated case-finding tool for frailty with good sensitivity and specificity. This study aimed to translate, culturally adapt, and validate the PRISMA-7 questionnaire for Chinese use.

**Methods:**

A prospective observational study with convenience sampling recruited bilingual adults aged 65 and over living in the community. The Functional Autonomy Measurement System (SMAF) was the gold standard benchmark. The English PRISMA-7 questionnaire was culturally adapted to Chinese using forward and backward translation. Intra- and inter-rater reliability were determined using the intraclass correlation coefficient (ICC). Face, content and criterion validity were determined. The Receiver Operator characteristic (ROC) curve determined the optimal cut-off score.

**Results:**

One-hundred-twenty participants (55 females and 65 males) were recruited. The Chinese PRISMA-7 questionnaire had excellent intra-rater and inter-rater reliability (ICC = 1.000). The rigorous forward and backward translation established the face and content validity. The moderately high correlations between the English PRISMA-7 with SMAF (*r* = − 0.655, *p* <  0.001) and Chinese PRISMA-7 with SMAF (*r* = − 0.653, *p* <  0.001) pairs established the criterion validity. An optimal cut-off score of three “Yes” responses was reported with 100% sensitivity and 85.3% specificity.

**Conclusion:**

This translation, cross-cultural adaptation, and validation study established the Chinese PRISMA-7 questionnaire. The preliminary results suggest adequate diagnostic test accuracy for frailty screening among the Chinese-literate community.

**Supplementary Information:**

The online version contains supplementary material available at 10.1186/s12877-024-04825-5.

## Introduction

Frailty is a medical syndrome characterised by diminished strength, endurance and reduced physiologic function [1], commonly brought upon by age-associated decline in functions across multiple organ systems [2]. Frailty entails an increased risk of adverse health outcomes such as functional decline [3, 4], falls, hospitalisation, weakness and fatigue, increased healthcare costs in community-dwelling older individuals, compromised quality of life and increased mortality [5–7]. O’Caoimh et al. (2020) estimated that the prevalence of pre-frailty and frailty were 46% and 12%, respectively, in 62 countries [8]. At the same time, the local estimated prevalence of pre-frailty and frailty were 37% and 6.2%, respectively [5]. Fortunately, frailty may be modifiable or is often modifiable with early detection [9] and can be managed through a comprehensive treatment plan comprising medications, physical activity, nutritional support and oral supplementation [10]. However, the successful management of frailty lies within early detection. Thus, effective early detection is crucial. Established clinical practice guidelines recommend that individuals above the age of 70 or younger individuals with unintentional loss of more than 5% of body weight in a year should receive routine screening for frailty [11]. Early identification of frail individuals allows for timely intervention and prevents dependency [1].

Choosing the right instrument is paramount in the early detection of frailty [12, 13]. However, there is no gold standard in frailty screening and detection. A 7-item questionnaire developed from the Programme on Research for Integrating Services for the Maintenance of Autonomy (PRISMA-7) was established to address a lack of continual care experienced by seniors with chronic conditions [14]. The PRISMA-7 comprises seven questions relating to the risk factors for frailty and is a simple tool to administer. It has good sensitivity and specificity of 78.3% and 74.7%, respectively [14], and is a validated tool to screen for frailty in individuals older than 75 [15]. The Asia-Pacific Clinical Practice Guidelines for the Management of Frailty and the British Geriatrics Society both have endorsed PRISMA-7 as part of the recommended tool to identify frailty, supported by studies that show the excellent performance of the questionnaire compared to other instruments with the same purpose [11, 16].

The PRISMA-7 questionnaire has been translated extensively into different languages, such as European Portuguese [17], Brazilian Portuguese [18], Turkish [19] and German [20]. To date, there is no validated Chinese-translated PRISMA-7. With approximately 1.1 billion speakers, the Chinese language is the second-most spoken language worldwide [21], and approximately 75% of Singaporeans are Chinese, with 48% having Chinese as their primary language [22]. Furthermore, according to the United Nations, by 2035, the percentage of the population older than 65 may rise to 28.5% in Hong Kong, 26.5% in Taiwan, 25.9% in Singapore, 20.7% in China and 11.5% in Malaysia, which are among the major Chinese-speaking countries merely within the Asia-Pacific region [23]. Despite the statistical presence of an alarming rate of Chinese-literate monolingual seniors, there has yet to be a cross-cultural adaptation and validation of the PRISMA-7 questionnaire appropriate for the Chinese-speaking population. With no proper standardisation of the delivery of instructions in Chinese, translation of the PRISMA-7 questionnaire may only be done on an ad-hoc basis, leading to substantial variations among clinicians and posing critical threats to the reliability of the survey results. The implication of ad-hoc translation is well-established [24]; paraphrasing may potentially allude to the essential and pivotal information directly or indirectly, leading to failure to account for accuracy, particularly in the unique healthcare setting. This highlights the need for Chinese instructions. Thus, this study aimed to: (1) translate, culturally adapt and validate the PRISMA-7 questionnaire in Chinese; (2) establish the reliability of the new Chinese PRISMA-7 questionnaire; (3) determine an optimal cut-off score for the Chinese PRISMA-7; (4) explore the relevance and applicability of the Chinese PRISMA-7 to the younger geriatric population age 65 to 74 for early frailty detection.

## Methods

### Setting and design

With guidance from the Strengthening the Reporting of Observational Studies in Epidemiology (STROBE) and Guidelines for Reporting Reliability and Agreement Studies (GRRAS) statements statement [25, 26], this study adopted the prospective observational study via convenience sampling with participants recruited from the community centres of the residential districts in Singapore. The study obtained ethical approval from the Singapore Institute of Technology - Institutional Review Board (SIT-IRB) (Approval number: 2021052). All methods were performed under the local guidelines and regulations from August 2021 to January 2022. All research participants voluntarily provided written informed consent for personally identifiable data, including biomedical and biometric data. Confidentiality was maintained throughout the study, and data analysis was performed only with anonymised data. Data collection took place in various community districts in Singapore. The originators of PRISMA-7 [14] consented and granted permission for the study.

### Participants

#### Sample size calculation

Based on the Singaporean population of 5.4 million [27] and the Cochran’s formula $n=\frac{Z^2}{e^2} pq$ was used to determine the sample size [28], where the *z*-value = 1.96; estimated proportion of the population, *p* = 0.062 [5]; *q* = (1- p) = (1 - 0.062) = 0.9938; the margin of error, *e* = 0.05. A minimum of 90 participants was required. We allowed for a possible attrition rate of 30%; thus, a minimum of 117 participants (*n* = 117) were needed.

#### Inclusion and exclusion criteria

Users of the community centres were conveniently recruited. The inclusion criteria were: (1) community-dwelling Chinese Singaporeans aged 65 and above; (2) ability to understand conversational/colloquial English and Chinese, or the presence of a proxy if the participant is unable to elicit a response [18]. Participants were excluded if they: (1) were unable to communicate in either English or Chinese; and (2) had no proxy to mitigate the communication issue.

### Development of the Chinese PRISMA-7 questionnaire

**Fig. 1:**
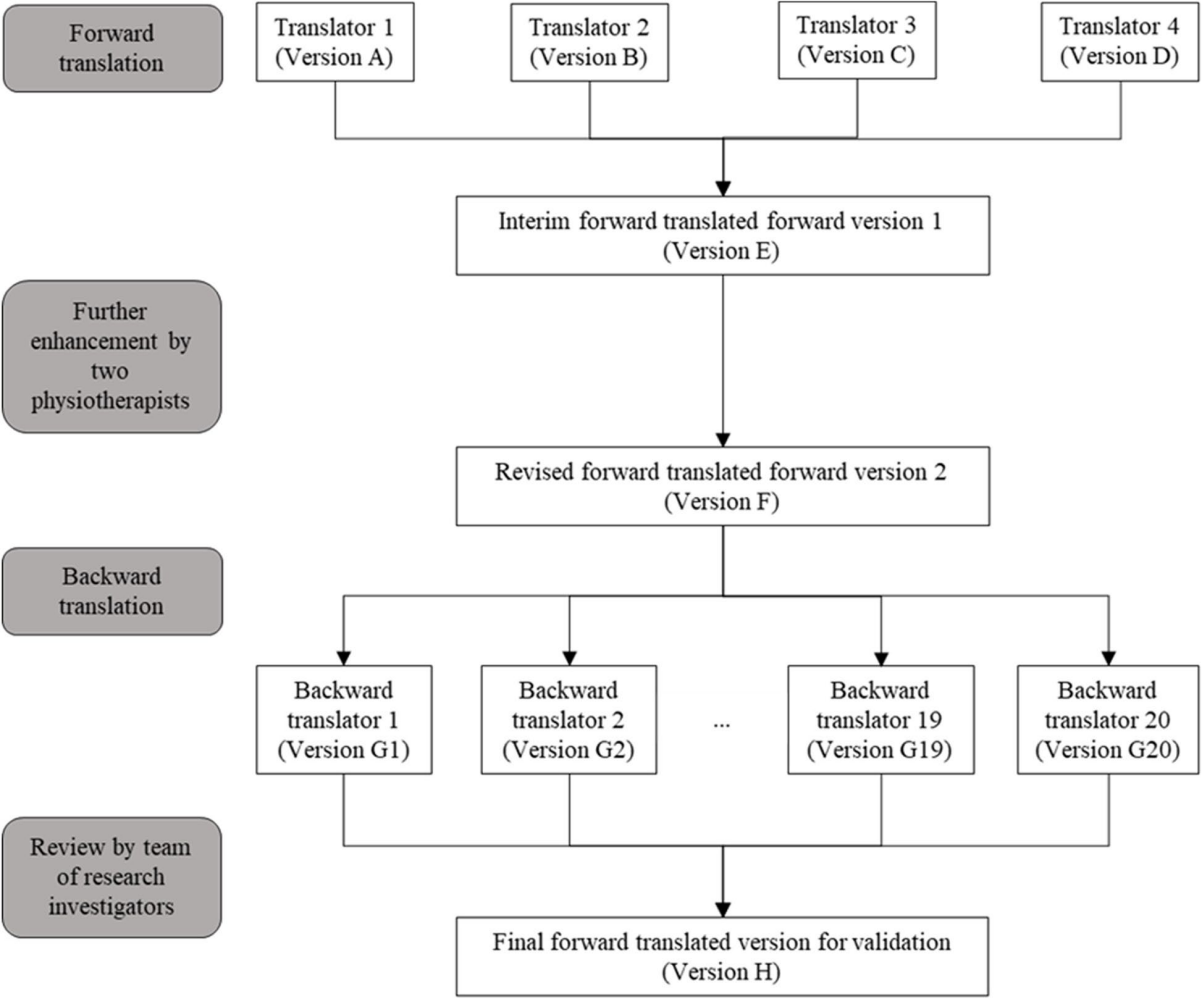
Flow diagram of the translation and cross-cultural adaptation process

The translation and cross-cultural adaptation process of the PRISMA-7 questionnaire were adopted from Brislin’s translation model and followed the guidelines provided by the American Association of Orthopaedic Surgeons [29, 30]. Figure 1 depicts the five-phased translation and cross-cultural adaptation process to develop the Chinese PRISMA-7 questionnaire. Four independent translators, fluent in English and Chinese and familiar with the PRISMA-7 questionnaire, performed the forward translation of the original questionnaire from English to Chinese (Versions A to D). The four versions were compared and referenced to synthesise the interim forward translated version E. Two physiotherapists, who were not involved with the initial forward translations, reviewed and adjusted the overall presentation of the interim questionnaire to formulate version F. Subsequently, the backward translation was performed from Chinese (version F) to English (Version G1 to G20), by 20 laypersons who were colloquially fluent in English and Chinese, but with no prior knowledge of the PRISMA-7 questionnaire. The 20 laypersons, aged 18 to 45 years, originated from diverse and non-healthcare backgrounds. Lastly, the panel of researchers, which consisted of four investigators and two physiotherapists, reviewed the 20 backwards translated versions for face and content validity. Modifications were made as necessary to produce the final Chinese PRISMA-7 questionnaire (Version H). The determination of face and content validity took place during the translation and developmental process of the Chinese PRISMA-7.

### Instrumentation

#### PRISMA-7

The original PRISMA-7 questionnaire consists of seven questions related to the risk factors for frailty [14]. Every “yes” response contributes to a point; an individual with more than three positive responses (3 “yes”) is considered at risk of frailty. A well-validated screening tool for frailty [15], it has good sensitivity (78.3%) and specificity (74.4%) [14].

#### Functional autonomy measurement system [Système de mesure de l’autonomie fonctionnelle (SMAF: abbreviation in French)]

The SMAF is a 29-item scale created following the WHO [31] classification of impairments [32–34]. It assesses five different aspects of functionality: activities of daily living (ADL) (7 items), mobility (6 items), communication (3 items), mental functions (5 items), and instrumental activities of daily living (IADL) (8 items). A maximum total score of − 87 is possible by scoring items on a negative 5-level scale ranging from 0 (independent), − 0.5 (independent but has difficulty carrying out the activity), − 1 (needs supervision or stimulation to carry out the activity, − 2 (needs some help to carry out the activity), − 3 (needs complete help to carry out the activity). A more negative score denotes a lower functional ability. The cut-off point of ≤ − 15 on the scale indicates moderate to severe loss of functional capacity [14]. The SMAF must be performed by a trained health professional that scores the actual performance of the individual after obtaining the information by questioning the subject and proxies, by observing and even evaluating the individual. The investigators of this study received training provided by Le Centre d’expertise en santé de Sherbrooke (CESS) to acquire the knowledge and skills and were all certified proficient in administering the SMAF before data collection. The SMAF scale has excellent test-retest and interrater reliability, with intraclass correlation coefficients (ICC) ranging from 0.95 for the overall score to 0.84 (mental functions) to 0.96 (ADL) for the subscores [35]. Several studies have assessed its validity [32–34, 36]. The SMAF was chosen as the gold standard for establishing the criterion validity in this study as it was the same instrument used to develop the original English PRISMA-7 [14].

### Data collection

#### Pilot trial

A pilot feasibility trial with 10 participants (*n* = 10) was conducted before the full-scale data collection to prepare the investigators for logistic, administrative, and procedural requirements [37, 38]. All participants met the same inclusion and exclusion criteria. The intra- and inter-rater reliability of the Chinese PRISMA-7 (Version H) and SMAF were also determined during this phase before the full study commenced with the four researchers (YG, SQT, KQL, HXL) on this study. All participants completed the English PRISMA-7 questionnaire once, Chinese PRISMA-7 and SMAF thrice, administered by random allocations of investigators on several occasions.

#### Full-scale study

The full-scale study proceeded with experience gathered during the pilot trial which enhanced the logistic and administrative workflow. Following the defined inclusion and exclusion criteria, community-dwelling individuals were recruited via convenience sampling at various districts in Singapore. The four researchers collected variables including age, gender and the need for a proxy (helper). Every participant completed the English and Chinese versions of the PRISMA-7 and the SMAF once within 2 days in randomised sequence, to prevent fatigue or learning effects from taking multiple questionnaires in quick succession. Frailty is generally considered to be relatively stable in the short term, especially in the absence of acute illness or significant changes in health status. It reflects long-term, cumulative effects of ageing and health conditions rather than short-term fluctuations. Therefore, it’s unlikely that the status of individuals in relation to the frailty screening would have significantly changed within the period between questionnaire administrations in this study. The scores of PRISMA-7 questionnaires, SMAF, and the demographic variables were used for statistical analysis. Standardised interview questions established during the pilot trial, constructed questionnaires (Chinese and English PRISMA-7 and SMAF), and established intra- and inter-rater reliability were among the measures used to address potential data sampling bias. The investigators were proficient with administering the English and Chinese PRISMA-7 and SMAF independently.

### Statistical analysis

Statistical analysis was performed using IBM SPSS Statistics for Windows, Version 28.0, with statistical significance set as *p* <  0.01. There was no missing data among the 120 participants. Descriptive statistics were used to determine the characteristics of the participants. The ICC determined the intra- and inter-rater reliability: the two-way mixed model established the intra-rater reliability, while the two-way random model examined the inter-rater reliability. Face validity was established during the translation process. Content validity was determined by the content validity index (CVI) and content validity ratio (CVR). CVI examines the relevance and clarity, and it can be calculated using the Item-CVI (I-CVI) and the Scale-CVI (S-CVI) [39]. The I-CVI was determined by the proportion of the number of raters in agreement. A panel of 6 investigators rated the seven translated items using a 4-point Likert scale (1 = irrelevant, 2 = needs major revision, 3 = needs minor revision and 4 = complete). The I-CVI was calculated by the total ratings scored by all panel members. The I-CVI is considered relevant if greater than 0.79; 0.70 to 0.79 requires revision; the item should be eliminated if less than 0.70 [39–41]. Similarly, the S-CVI evaluates the number of items in the instrument that received a “highly complete” grade. The Universal Agreement (UA) among the panel members (S-CVI/ UA) and the Average CVI (S-CVI/Ave) are two ways of determining S-CVI, the latter being a less conservative method [39]. S-CVI/UA is calculated by the sum of all items with I-CVI equal to 1 divided by the total number of items, while the Average S-CVI (S-CVI/Ave) is calculated by dividing all the I-CVIs by the number of items. Content validity is excellent when S-CVI/UA is more than 0.8, and the S-CVI/Ave is more than 0.90 [41]. CVR measures the essentiality of an item [42] and ranges from − 1 to 1, with a higher score indicating a greater agreement among panel members. The 3-point Likert scale (1 = not essential, 2 = useful but not essential, and 3 = essential) determined the essentiality of each of the seven questions in the Chinese PRISMA-7, with ratings performed by the same 6-investigator panel. The calculation of CVR used the following formula: CVR = $\frac{n-\left(\frac{N}{2}\right)}{\frac{N}{2}}$, where n is the number of panellists who rated an item as “essential” and N is the total number of panellists [39]. For a panel size of six, CVR = 1.0 (*p* <  0.05) was required to be statistically significant [43]. Internal consistency, or the degree to which an instrument’s components are correlated, was evaluated using Cronbach’s alpha. Correlations were calculated between the individual item scores and the overall item score. Subsequently, factor analysis with principal component analysis (PCA) was used to examine if the items represented the construct to be measured [44]. Criterion validity was analysed using Pearson’s correlation coefficient (r). The correlations between the Chinese PRISMA-7 with the SMAF and the English PRISMA-7 with the SMAF determined the criterion validity. The sensitivity and specificity of the different cut-off scores were examined. The receiver operating characteristic (ROC) curves were constructed and appraised to determine and compare the optimal cut-off scores for the Chinese PRISMA-7 with the original English version.

## Results

### Chinese PRISMA-7

Table 1 presents the version of the Chinese translation [written in simplified or traditional Chinese (Note: there are no cultural, sentence-structural or grammatical variations between the two written forms and can be considered interchangeable)] established in this study.

**Table 1:** Translation of the PRISMA-7 questionnaire and version used for validation

	Original English	Simplified Chinese	Traditional Chinese
	Question	问题	問題
	Answer: Yes; No	回答: 是; 否	回答: 是; 否
1.	Are you 85 years old or older?	你是否超过85岁?	你是否超過85歲?
2.	Male?	男性?	男性?
3.	In general, do you have any health problems that require you to limit your activities?	平日, 你是否有会限制你日常活动的健康问题?	平日, 你是否有會限制你日常活動的健康問題?
4.	Do you need someone to help you on a regular basis?	你是否经常需要他人帮助?	你是否經常需要他人幫助?
5.	In general, do you have any health problems that require you to stay at home?	平日, 你是否有因为任何健康或疾病问题而需要待在家里减少外出?	平日, 你是否有因為任何健康或疾病問題而需要待在家裡減少外出?
6.	In case of need, can you count on someone close to you?	当你需要协助时, 是否有人可以依靠?	當你需要協助時, 是否有人可以依靠?
7.	Do you regularly use a cane, a walker or a wheelchair to move about?	你是否经常需要使用拐杖, 助行器或轮椅等辅助器材步行?	你是否經常需要使用拐杖, 助行器或輪椅等輔助器材步行?

### Reliability

The intra- and inter-rater reliability of the four researchers, with three independent replicate observations per participant, on Chinese PRISMA-7 was 1.0 (Table 2), indicating excellent reliability with the investigators obtaining identical results from participants due to the simplistic structure of the questionnaire. Similarly, the reliability of SMAF was excellent: the intra-rater reliability was 0.991 [95% confidence interval (95% CI): 0.964 – 0.998, *p* < 0.001], and the inter-rater reliability was 0.973 (95% CI: 0.901 – 0.993, *p* < 0.001) (Table 3).

**Table 2:** Reliability of the Chinese PRISMA-7 questionnaire

		95% CI^a^	
Chinese PRISMA-7	ICC^b^ value	Lower bound	Upper bound
Intra-rater reliability single measure	1.000	.	.
Inter-rater reliability single measure	1.000	.	.

^a^*CI* Confidence Interval, ^b^*ICC* Intra-class Coefficient

**Table 3:** Reliability of the SMAF

		95% CI^a^		
SMAF^b^	ICC^c^ value	Lower bound	Upper bound	*p*-value
Intra-rater reliability single measure	0.991	0.964	0.998	< 0.001
Inter-rater reliability single measure	0.973	0.901	0.993	< 0.001

^a^*CI* Confidence Interval, ^b^*SMAF* Functional Autonomy Measurement System, ^c^*ICC* Intra-class Coefficient

### Validity

**Fig. 2:**
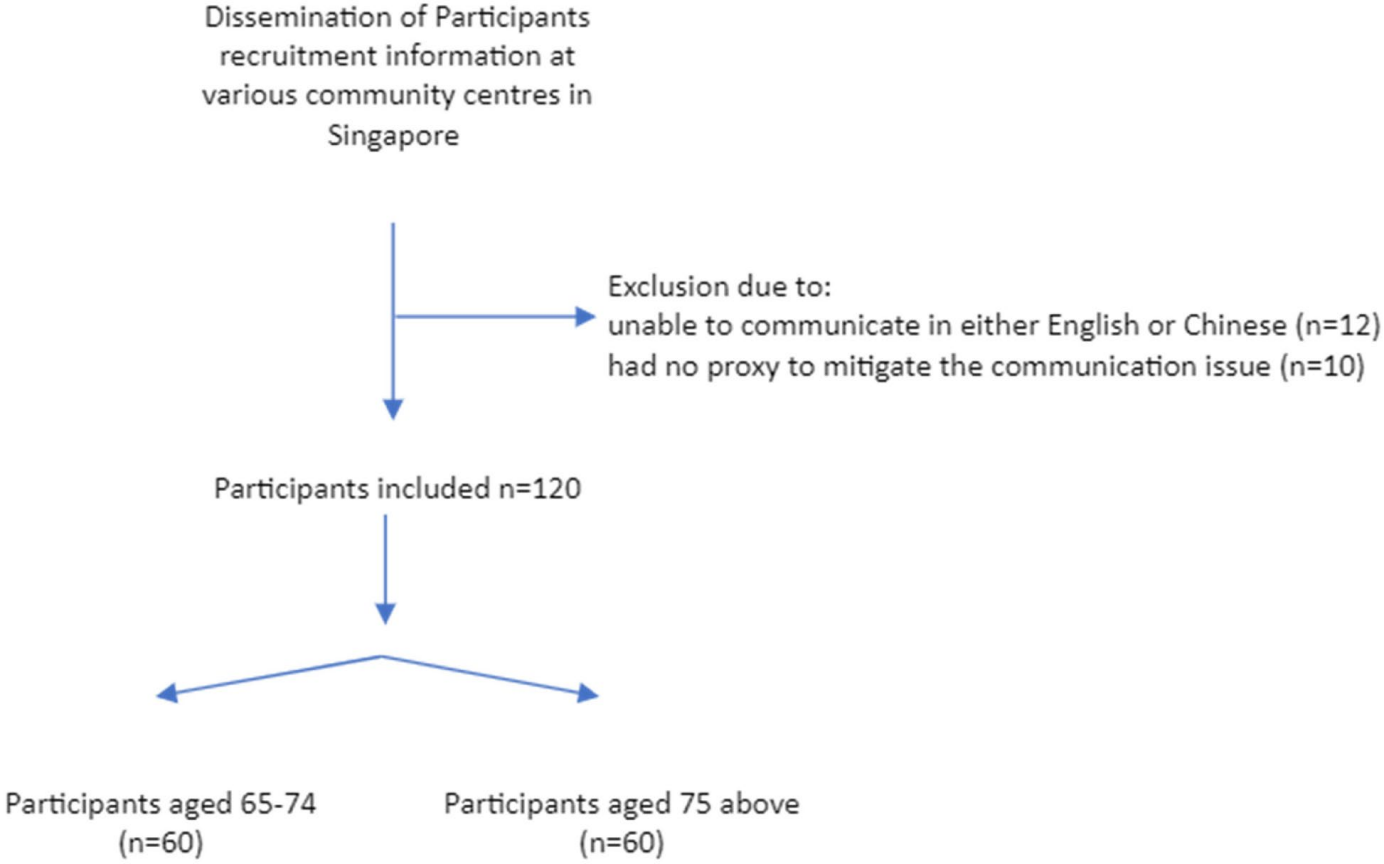
Participant recruitment flowchart

This validity study recruited a total of 120 participants (*n* = 120) (Fig. 2). Table 4 presents the distribution by gender and age of the recruited participants and the distribution of the Chinese PRISMA-7 scores, while Table 5 reports SMAF score comparisons against the Chinese PRISMA-7. No participants withdrew from the study, and no missing data was found from all the return questionnaires. Forty-one participants required the use of the proxy responders during data collection. The proxy responder is a person, a family member or a caregiver in the context of this study who answered the PRISMA-7 and SMAF questionnaires on behalf of the participants due to reasons such as cognitive impairment or severe illness [14, 34] an acceptable practice with both questionnaires.

**Table 4:** Number of positive responses to the questionnaire, strata zero (P0) to seven (P7) against gender and age

PRISMA	Variable				
	Gender		Age (Years)		
	Female	Male	65-74	75-84	Above 85
P0 (%)	3 (2.5)	0 (0.0)	2 (1.7)	1 (0.8)	0 (0.0)
P1 (%)	34 (28.3)	8 (6.7)	24 (20.0)	16 (13.3)	2 (1.7)
P2 (%)	5 (4.2)	48 (40.0)	32 (26.6)	19 (15.9)	2 (1.7)
P3 (%)	9 (7.5)	6 (5.0)	2 (1.7)	8 (6.7)	5 (4.1)
P4 (%)	3 (2.5)	2 (1.7)	0 (0.0)	2 (1.7)	3 (2.5)
P5 (%)	1 (0.8)	1 (0.8)	0 (0.0)	1 (0.8)	1 (0.8)
P6 (%)	0 (0.0)	0 (0.0)	0 (0.0)	0 (0.0)	0 (0.0)
P7 (%)	0 (0.0)	0 (0.0)	0 (0.0)	0 (0.0)	0 (0.0)
Total	55 (45.8)	65 (54.2)	60 (50%)	47 (39.2)	13 (10.8)

**Table 5:** Demographic data, Chinese PRISMA-7 and SMAF scores of participants (*n* = 120)

**Age**	**Gender**	***n***	**Gender**	***n***
65 – 74	Male	37	Female	23
≥75	Male	30	Female	30
	**No. of Participants (*n*)**	**Chinese PRISMA-7 Score**	**SMAF Positive (≤ − 15 points)**	**SMAF Negative (> − 15 points)**
65 - 74	2	0	0	2
	21	1	0	21
	32	2	0	32
	4	3	0	4
	0	4	0	0
	0	5	0	0
	1	6	1	0
	0	7	0	0
**Total**	**60**		**1**	**59**
≥75	1	0	0	1
	14	1	0	14
	22	2	0	22
	12	3	2	10
	6	4	3	3
	3	5	3	0
	2	6	2	0
	0	7	0	0
Total	**60**		**10**	**50**

Face validity was determined by the 20 different individuals conducting the backward translation of the Chinese PRISMA-7 questionnaire back to English. All investigators agreed that the Chinese PRISMA-7 questionnaire had met its intended purpose. All seven items on the Chinese PRISMA-7 have a CVR of 1.00. Similarly, all items obtained an I-CVI of 1.00, except for item 5, with a score of 0.83. The S-CVI/UA was 0.86, and S-CVI/Ave was 0.98. The Cronbach’s alpha coefficient [45] for internal consistency was 0.515. However, excluding item 2 increased Cronbach’s alpha to 0.680. Table 6 presents the correlation matrix of the instrument items. The parallel analysis indicated the presence of two eigenvectors (factors). The first three eigenvalues from the correlation matrix were 2.65, 1.14 and 0.99. These two factors explained 54.3% of the total variance of the data set. Table 7 represents the factor loading of the 7 items from the two factors. The PCA biplot indicated that items 1, 3, 4, 5 and 7 correlated well.

**Table 6:** Correlation matrix of variables

Items	Item 1	Item 2	Item 3	Item 4	Item 5	Item 6	Item 7
Item 1	1.000	−0.166	0.117	0.245	0.161	0.097	0.466
Item 2	−0.166	1.000	−0.185	− 0.151	− 0.278	− 0.166	− 0.216
Item 3	0.117	− 0.185	1.000	0.528	0.452	−0.13	0.454
Item 4	0.245	−0.151	0.528	1.000	0.402	0.035	0.582
Item 5	0.161	−0.278	0.452	0.402	1.000	0.110	0.223
Item 6	0.097	−0.166	− 0.13	0.035	0.110	1.000	0.073
Item 7	0.466	−0.216	0.454	0.582	0.223	0.073	1.000

**Table 7:** Factor loadings of items

Items	Factors	
	1	2
1	0.514	−0.282
2	−0.414	0.522
3	0.727	0.346
4	0.799	0.249
5	0.641	−0.002
6	0.154	−0.778
7	0.788	0.040
Variance	0.380	0.543

The comparably high negative correlations were observed from the Pearson’s correlation coefficients between the English PRISMA-7 with SMAF [all participants (*r* = − 0.655), aged 65-74 (*r* = − 0.426) and aged 75 and above (*r* = − 0.696), *p* < 0.001] and Chinese PRISMA-7 with SMAF [all participants (*r* = − 0.653), aged 65-74 (*r* = − 0.441) and aged 75 and above (*r* = − 0.700), *p* < 0.001] (Table 8). Thus, the established criterion validity is inferred.

**Table 8:** Comparisons of *r* values of the English PRISMA-7 questionnaire, the Chinese PRISMA-7 questionnaire and the SMAF

Criterion Validity				
		English PRISMA-7 (95% CI)	Chinese PRISMA-7 (95% CI)	
SMAF	All participants	−0.655 (− 0.746 to − 0.539)	−0.653 (− 0.745 to − 0.537)	*p* < 0.001
	*Subgroup*			
	65 – 74 years	−0.426 (− 0.613 to − 0.193)	−0.441 (− 0.625 to − 0.210)	
	≥ 75 years	−0.696 (− 0.807 to − 0.537)	−0.700 (− 0.810 to − 0.543)	

*95%CI* 95% confidence interval, *p* < 0.01

**Fig. 3:**
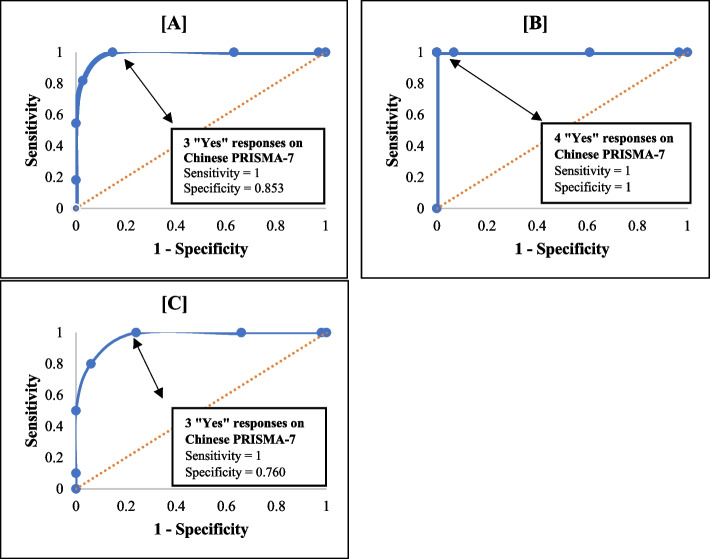
Receiver Operator Characteristic (ROC) Curves. *Notes:* (**A**) ROC curve for all participants, (**B**) ROC curve for participants aged 65 to 74 years, (**C**) ROC curve for participants aged 75 and above

Tables 9, 10 and 11 show all the possible cut-off points for the Chinese PRISMA-7 questionnaire for all participants, participants aged 65 to 74, and participants aged 75 and above, respectively, as well as their respective sensitivities, specificities, and positive and negative predictive values. The graphical relationships between sensitivity and specificity by the Receiver Operating Characteristic (ROC) curves are in Fig. 3.

**Table 9:** Characteristics of the possible cut-off scores for the chinese PRISMA-7 questionnaire for all participants

Cut-off	Sensitivity (%)	Specificity (%)	Positive predictive value (%)	Negative predictive value (%)
0	100	0	9.0	–
1	100	2.8	9.0	100
2	100	36.7	14.0	100
**3**	**100**	**85.3**	**41.0**	**100**
4	81.8	97.2	75.0	98.1
5	54.5	100	100	95.6
6	18.2	100	100	92.4
7	0.0	100	–	90.8

- undefined. Optimal cut-off score in bold

**Table 10:** Characteristics of the possible cut-off scores for the chinese PRISMA-7 questionnaire for participants aged 65 to 74

Cut-off	Sensitivity (%)	Specificity (%)	Positive predictive value (%)	Negative predictive value (%)
0	100	0	2.0	–
1	100	3.4	2.0	100
2	100	39.0	3.0	100
3	100	93.2	20.0	100
**4**	**100**	**100**	**100**	**100**
5	100	100	100	100
6	100	100	100	100
7	0.0	100	–	98.3

- undefined. Optimal cut-off score in bold

**Table 11:** Characteristics of the possible cut-off scores for the chinese PRISMA-7 questionnaire for participants aged 75 years and above

Cut-off	Sensitivity (%)	Specificity (%)	Positive predictive value (%)	Negative predictive value (%)
0	100	0.0	16.7	–
1	100	2.0	17.0	100
2	100	34.0	23.0	100
**3**	**100**	**76.0**	**45.0**	**100**
4	80.0	94.0	73.0	96.0
5	50.0	100	100	90.9
6	10.0	100	100	84.7
7	0.0	100	–	83.3

- undefined. Optimal cut-off score in bold

## Discussion

This study aimed to translate, culturally adapt, and validate the PRISMA-7 questionnaire in Chinese. The intra- and inter-rater reliability of the Chinese PRISMA-7 were very high, with no disagreement within and between raters. It is comparable to the similarly high ICC of the intra- and inter-rater reliability of the SMAF, proving its reliability and strongly suggesting that the instrument is suitable for use by non-healthcare individuals, ready for community screening and utilisation after simple training.

The rigorous process adopted from Brislin’s (1970) translation model and the five-phased translation process [29] established the face validity of the Chinese PRISMA-7, while the high CVI and CVR established the content validity. The analysis of the psychometric properties of the Chinese PRISMA-7 was congruent with the original study [14]. Internal consistency, evaluated using Cronbach’s alpha, was only 0.515, possibly due to the sensitivity of the statistic concerning the number of items on the instrument [46], particularly in the case of the 7-item PRISMA-7 questionnaire. On the other hand, the exclusion of item 2 increased Cronbach’s alpha to an acceptable level (α = 0.680) [47]. A similar trend was previously reported in the Brazilian version of the PRISMA-7 [18]. Factor analysis indicated the existence of two factors. The first factor explained 38% of the total variance, whereas the second elucidated 54.3%. Items 1, 3, 4, 5 and 7 displayed greater consistency with factor 1, with Item 6 of a lesser loading. Item 2 (male gender) alone contributed 52.2% of the 54.3% variance with factor 2 (Table 7).

The Pearson’s correlations demonstrated a congruent pattern when comparing the Chinese PRISMA-7 and SMAF with the English PRISMA-7 and SMAF pairs. For all participants and subgroup aged 75 years and above, both the pairs had similar negative moderate relationships with the SMAF (*r* values ranged from − 0.700 to − 0.653, all *p* < 0.001) (Table 8). Weak negative relationships were consistently observed between the subgroup aged 65 to 74 (*r* values ranged from − 0.441 to − 0.426, all *p* < 0.001). Notably, the original PRISMA-7 is validated only for those aged 75 and above. However, the Chinese PRISMA-7 is being validated for those between 65 to 74 years old in the current study. Overall, the profile of the participants recruited in this study showed diversity in age and gender, implying the applicability of the questionnaire within the Chinese-literate population.

The cut-off score for the Chinese PRISMA-7 questionnaire agrees with the original score [14] and the German translation report [20]. Overall, with all participants and subgroup aged 75 years and above, the optimal cut-off score of three or more “Yes” responses on the Chinese PRISMA-7 questionnaire coincides with the English and German PRISMA-7 questionnaire. A cut-off score of three “Yes” on the original questionnaire yielded the highest sensitivity of 78.3% and specificity of 74.7% [14], whereas the German translation reported a 100% sensitivity and 80% specificity [20]. The current study established a 100% sensitivity and 85.3% specificity from all participants, along with 100% sensitivity and 76% specificity from the subgroup aged 75 and above, inferring that only 14.7% of individuals without the risk may be incorrectly identified. Another interesting finding of this study was the different cut-off scores for those aged 65 to 74. An optimal cut-off score of four “Yes”, would yield the highest sensitivity and specificity instead of the score of three. The optimal cut-off score of 4 was similarly reported for other translation attempts, namely the Turkish (sensitivity = 81.5% and specificity = 88.2%) [19] and Brazilian Portuguese (sensitivity = 74.4% and specificity = 80%) [18] translations. The varied cut-off scores for participants aged 65 to 74 and those above 75 may be attributed to the two groups’ different health statuses and accessibility to primary health services. In recent times, most individuals in their early older adult years are more active and in better health than their forefathers [48]. Indeed, it was the case with the recruited participants of this study. From the inspection of the raw data (Table 5), only 1 out of the 60 participants aged 65-74 scored less than − 15 on SMAF compared to 10 out of the 60 participants from the above 75 group were presented with moderate to severe loss of functional capacity. The 11 participants in this study identified with frailty of the sample size of 120 equated to 9.2% frailty prevalence, identified by the SMAF. A similar trend of the positive responses of the PRISMA-7 (Tables 4 & 5) also revealed that no participants from the group aged 65-74 scored more than 4 “yes” in this current study. While the recruitment of all the participants (both age groups) took place simultaneously throughout the study period with no pre-screening mechanism, this concurrent and standardised convenience sampling of community-dwelling older adults in various residential districts of Singapore could not curb this sampling inequivalence. It is observed that the recruited participants from the group aged 65-74 were more functionally capable than their older counterparts despite all being community-dwelling individuals. Therefore, we postulate this is the main underlying contribution to the lack of power pointing out the different cut-off score for the younger sub-group via the ROC curve. After all, it is logically congruent with the idea that functional decline is age-related [2]. Frailty prevalence varies with the assessment tool, population, age, and location. Compared to earlier studies reporting 6.2% in Singapore [5], our findings show a slightly higher prevalence, but lower than 11% in Asia [8]. It may be an indication of a need for another study involving more participants presenting the condition (moderate to severe functional decline) in the age group 65-74 years, meaning a larger sample. In terms of mass screening [49], it may also indicate that case-finding may be questioned for priority in the age group 65-74 years since the condition is much less prevalent than in the age group 75 and over.

Some limitations in this study should be highlighted. While this study intended to explore the relevance and applicability of the Chinese PRISMA-7 in the young geriatric population (aged 65 to 74), the subgroup sample size of 60 and those with less apparent functional decline may not have provided sufficient statistical power. This could be one of the attributing factors for the cut-off score of four, yielding a 100% sensitivity and specificity with the ROC curve analysis. However, it is impossible to conclude such results in reality confidently. Future studies should be conducted with a larger sample size or include more participants with moderate to severe functional decline further to explore the relationship between age and psychometric properties. Additionally, it’s important to note that we did not have access to information regarding the education or socio-economic status of the participants. Despite the missing information which could potentially influence comprehension, these frailty screening tools require minimal linguistic ability and understanding from the participants, making them suitable for widespread application. In spite of this, the current study will serve as a foundation to investigate the validity of the Chinese PRISMA-7 questionnaire among the Chinese-literate young geriatric population. Additionally, despite increasing global popularity in frailty screening and prevention, the limited availability of validated Chinese frailty screening tools curbs the comparability of the current PRISMA-7 questionnaire. However, the rigorous process of translation, cross-cultural adaptation, and validation lends overall confidence to the results.

## Conclusions

Identifying older individuals who are functionally dependent is critical for planning any integrated interventions in health services that emphasise prevention, care, and rehabilitation. Effective screening and early detection with sensitive and specific screening tools curtail frailty management. The PRISMA-7 cross-cultural adaptation to Chinese aimed to provide a simple tool that can be applied quickly and easily. This tool will help detect community older individuals with functional loss at the primary care entry point. The satisfactory psychometric properties in this study suggest the validity process was adequate, and the Chinese PRISMA-7 questionnaire is recommended to detect community-dwelling individuals with functional loss.

### Supplementary Information

**Supplementary Material 1.**

**Supplementary Material 2.**

## Data Availability

De-identifiable data and study materials used and/or analysed during the current study are available from the corresponding author upon reasonable request.

## Abbreviations

PRISMA-7: 7-item Programme on Research for Integrating Services for the Maintenance of Autonomy
SMAF (abbreviation in French): Functional autonomy measurement system (Système de mesure de l’autonomie fonctionnelle)
ADL: Activities of daily living
IADL: Instrumental activities of daily living
CESS (abbreviation in French): Le Centre d’expertise en santé de Sherbrooke
ICC: Intraclass correlation coefficients
CVI: Content validity index
CVR: Content validity ratio
I-CVI: Item-CVI
S-CVI: Scale-CVI
UA: Universal agreement
PCA: Principal component analysis
ROC: Receiver operating characteristic
